# The Effect of Ultrasound Image Pre-Processing on Radiomics Feature Quality: A Study on Shoulder Ultrasound

**DOI:** 10.1007/s10278-025-01421-w

**Published:** 2025-02-06

**Authors:** Matthaios Triantafyllou, Evangelia E. Vassalou, Alexia Maria Goulianou, Theodoros H. Tosounidis, Kostas Marias, Apostolos H. Karantanas, Michail E. Klontzas

**Affiliations:** 1 Artificial Intelligence and Translational Imaging (ATI) Lab, Department of Radiology, School of Medicine, University of Crete, Voutes Campus, Heraklion, Greece; 2 Department of Medical Imaging, University Hospital of Heraklion, Heraklion, Crete Greece; 3 Department of Orthopaedic Surgery, Medical School, University of Crete, 71110 Heraklion, Crete Greece; 4 Computational BioMedicine Laboratory, Institute of Computer Science, Foundation for Research and Technology (FORTH), Heraklion, Crete Greece; 5 Department of Electrical and Computer Engineering, Hellenic Mediterranean University, Heraklion, Crete Greece; 6 Division of Radiology, Department for Clinical Science, Intervention and Technology (CLINTEC), Karolinska Institutet, Stockholm, Sweden

**Keywords:** Radiomics, Ultrasound, Shoulder, Reproducibility, Image pre-processing

## Abstract

Radiomics, the extraction of quantitative features from medical images, has shown great promise in enhancing diagnostic and prognostic models, particularly in CT and MRI. However, its application in ultrasound (US) imaging, especially in musculoskeletal (MSK) imaging, remains underexplored. The inherent variability of ultrasound, influenced by operator dependency and various imaging settings, presents significant challenges to the reproducibility of radiomic features. This study aims to identify whether commonly used image pre-processing methods can increase the reproducibility of radiomics features, increasing the quality of analysis. This is performed with shoulder calcific tendinopathy as a case study. Ultrasound images from 84 patients with rotator cuff calcifications were retrospectively analysed. Three pre-processing techniques—Histogram Equalization, Standard CLAHE, and Advanced CLAHE—were applied to adjust image quality. Manual segmentation of calcifications was performed, followed by the extraction of 849 radiomic features. The reproducibility of these features was assessed using the intraclass correlation coefficient (ICC), comparing results across pre-processing methods within the dataset. The Advanced CLAHE pre-processing method consistently yielded the highest ICC values, indicating superior reproducibility of radiomic features compared to other methods. Wavelet-transformed features, particularly in the GLCM and GLRLM subgroups, demonstrated robust reproducibility across all pre-processing techniques. Shape features, however, continued to show lower reproducibility. Advanced CLAHE pre-processing significantly enhances the reproducibility of radiomic features in ultrasound imaging of calcifications. This study underscores the importance of pre-processing in achieving reliable radiomic analyses, particularly in operator-dependent imaging modalities like ultrasound.

**Supplementary Information:**

The online version contains supplementary material available at 10.1007/s10278-025-01421-w.

## Introduction

Radiomics, which involves the extraction of quantitative features from medical images, has the potential to significantly enhance diagnostic and prognostic models in clinical practice. However, while radiomics has been extensively explored in modalities such as CT and MRI, its application in ultrasound (US) imaging remains limited, particularly within musculoskeletal (MSK) imaging. Ultrasound has become a valuable tool for evaluating complex shoulder conditions, such as subacromial impingement syndrome [1]. Additionally, ultrasound radiomics has demonstrated superior diagnostic performance over traditional evaluations in conditions like carpal tunnel syndrome, highlighting its potential for robust and reproducible image analysis [2]. A major challenge in this area is the reproducibility of radiomic features [3, 4]. The inherent variability of ultrasound, influenced by factors such as operator dependency, the use of different probes, and various on-the-fly settings (e.g., depth, B-mode gain), can lead to inconsistencies in image acquisition, thereby undermining the reliability of radiomics in this modality [5, 6].

One area of particular interest in musculoskeletal ultrasound is the diagnosis of calcific tendinopathy of the rotator cuff tendons. Calcific tendinopathy is a common condition characterized by the deposition of calcium within the tendons, often leading to pain and functional impairment [7, 8]. Despite its prevalence, the radiomic analysis of calcifications has been largely overlooked. Given the variety of calcification texture ranging from soft to hard, radiomics could provide detailed insights into the characteristics of these calcifications that are not readily apparent through traditional imaging assessments.

Currently, discussions on pre-processing in ultrasound radiomics primarily focus on conventional methods like voxel resizing and intensity standardization [9]. These factors can be tuned at the extraction step, by configuring the extraction parameter file as in the case of Radiomics [10]. There is a growing interest in the use of image pre-processing methods to improve the stability of radiomic features. Techniques such as histogram equalization have the potential to enhance the robustness of radiomics by standardizing the image input before feature extraction. Unlike imaging modalities such as computed tomography or positron emission tomography, where intensity values directly reflect specific tissue characteristic, ultrasound lacks a standardized quantitative basis for its intensity values [11]. This makes it especially challenging to ensure feature reproducibility in ultrasound imaging, particularly for calcifications, as the effectiveness of these methods remains underexplored.

The aim of this study is to explore how various image pre-processing techniques affect the stability and reproducibility of radiomic features in musculoskeletal ultrasound imaging. The objective is to determine whether these methods can enhance the reliability of radiomics, increasing the reproducibility of radiomics features. In summary, while radiomics holds great promise for advancing ultrasound imaging—especially in the assessment of calcified structures—significant challenges remain in ensuring the reproducibility of radiomic features. Exploring the impact of image pre-processing methods on feature stability is a critical step toward realizing the full potential of radiomics in this field [12].

## Materials and Methods

### Dataset

This study retrospectively included 84 consecutive patients diagnosed with rotator cuff tendon calcification between January 2017 and March 2019 at the General Hospital of Sitia. Patients with calcifications that had unclear borders, those with tendon tears, or those with incomplete clinical data or ultrasound images were excluded. Ultrasound examinations were performed by an experienced radiologist (E.E.V.) with 10 years of expertise, using a GE Logiq F8 ultrasound machine equipped with a 6–12 MHz probe, in accordance with the European Society of Musculoskeletal Radiology’s technical guidelines [13]. Additionally, detailed information about the calcifications, including their maximum diameter, location, and hardness (classified as hard with acoustic shadowing, soft without shadowing, or fluid if an anechoic component was present), along with patient demographics such as age and sex, were documented.

To ensure patient confidentiality and comply with data protection regulations, all personal identifiers were removed from the clinical data and image metadata. Specifically, patient names were deleted, and each data set was assigned a unique consecutive number for identification purposes. The study was carried out in accordance with the Declaration of Helsinki and received approval from the Institutional Review Board of the University Hospital of Heraklion (Ref. No. 28107/05–12–2023). As the data collection was retrospective, the requirement for patient consent was waived.

### Image Pre-Processing

For image pre-processing, three different techniques were applied to enhance the ultrasound images: histogram equalization, a Standard CLAHE (Contrast-Limited Adaptive Histogram Equalization), and an Advanced CLAHE variation.

The first method, histogram equalization, enhances image contrast by distributing the most frequent intensity values across the entire grayscale spectrum. While this method can significantly improve the overall visibility of features, as a global technique, it may not sufficiently address finer, localized details within ultrasound images. This limitation can result in over-enhancement of certain regions, leading to artifacts or loss of important diagnostic information [14]. On the other hand, CLAHE, with its localized approach, offers more precise contrast enhancements by processing smaller image regions independently, with several applications in radiology [15, 16].

Subsequently, the images were processed using CLAHE with a clip limit of 2.0 and a fixed tile grid size of 8 by 8 pixels. This configuration, referred to as Standard CLAHE, is generally considered as default settings for non-medical tasks [17]. This method provides localized contrast enhancement by dividing the image into small regions (tiles) and applying histogram equalization to each one. Although this approach can lead to more pronounced contrast improvements, the higher clip limit may also increase the likelihood of introducing noise into the image [18]. By including this default CLAHE configuration, we aimed to explore its potential impact as a crude image enhancement technique and establish a baseline for comparison with more refined pre-processing methods commonly used in medical imaging applications.

Finally, a more sophisticated CLAHE technique was employed, featuring a lower clip limit of 0.9 and a dynamically calculated tile grid size set to 64 pixels, named Advanced CLAHE. This method offers a more adaptive contrast enhancement, aiming to provide subtler, image-specific improvements while minimizing the risk of noise and artifacts [19]. Unlike Standard CLAHE, which uses a fixed tile grid size of 8 × 8 pixels and a higher clip limit of 2.0, Advanced CLAHE adjusts its parameters dynamically to better account for the inherent variability in ultrasound images, enhancing fine details while controlling for noise. These differences make Advanced CLAHE particularly suited to the complex textures often encountered in medical imaging.

To evaluate the computational efficiency of the pre-processing methods, we processed a set of 10 cases with 20 repetitions per image. All pre-processing was conducted using Python version 3.12.1 on a Jupiter notebook running macOS Ventura (version 13.0) with 8 GB RAM on an Apple M2 8-core processor, and the code for the three pre-processing methods described can be found on GitHub https://github.com/mattria/Image-preprocessing-for-Ultrasound-Radiomics/tree/main.

### Segmentation and Feature Extraction

Manual segmentation of intratendinous calcific deposits was conducted by two radiology residents (M.T. and A.M.G.) with 3 years and 1 year of experience in segmentation tasks, respectively. The segmentation process was straightforward in most cases due to the distinct borders of calcifications. The senior researcher (BLINDED), with over 10 years of expertise in AI, musculoskeletal research, and segmentation, visually reviewed the segmentation masks for all cases to ensure consistency in adherence to the predefined segmentation criteria. The segmentation process was performed manually using the paintbrush tool in 3D Slicer software (version 5.2 for MacOS, last accessed on August 25th, 2024) [20]. The segmentation mask was built directly on the original images to preserve the natural representation of calcification borders, ensuring accuracy and consistency across all segmentations while reducing the potential for artifacts introduced by pre-processing methods. The same segmentation mask was applied to the three enhanced versions of each image (Histogram Equalization, Standard CLAHE, and Advanced CLAHE), as described in the previous subsection.

**Fig. 1:**
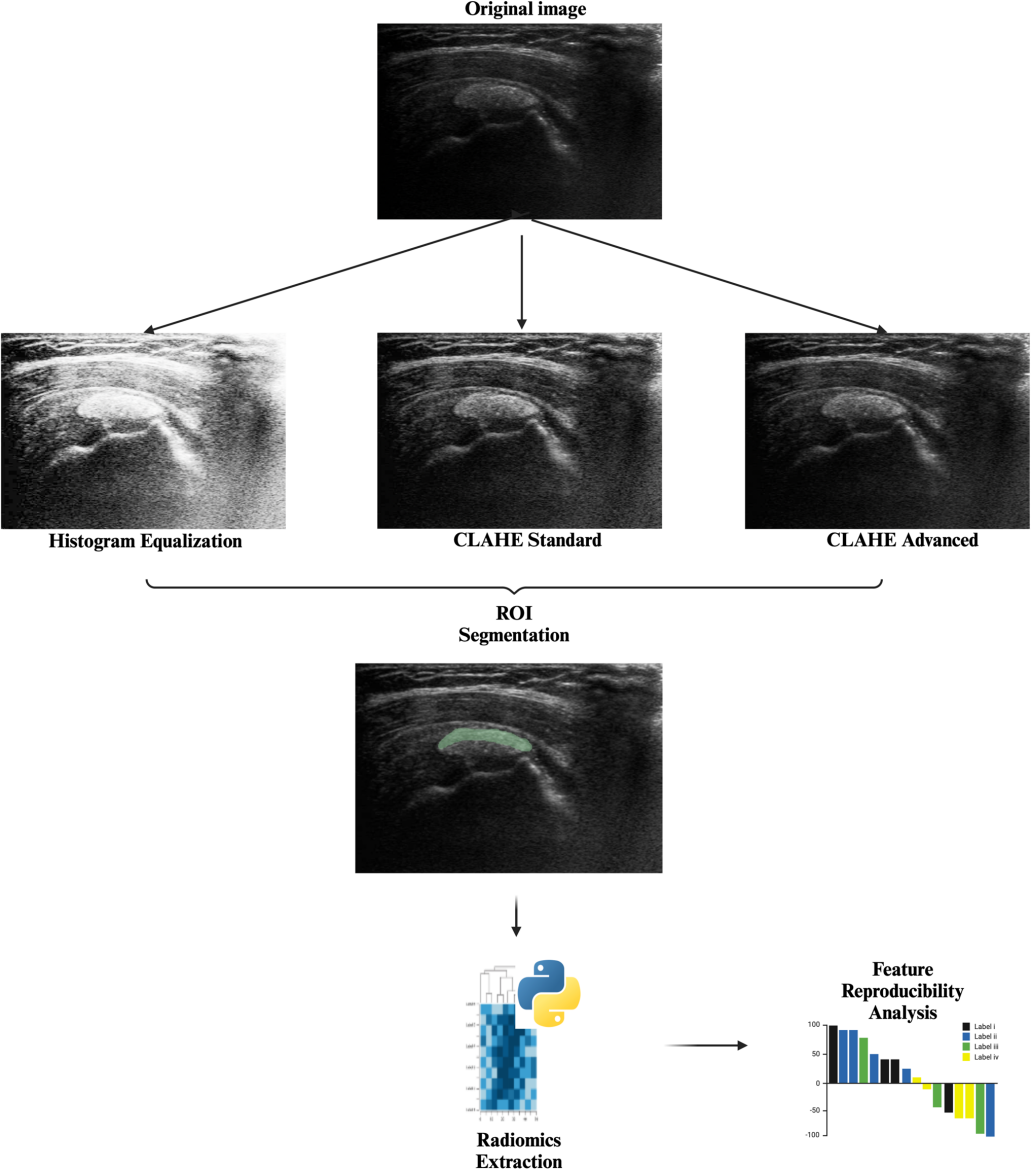
Flowchart of the study demonstrating image preprocessing, RoI segmentation, and data analysis steps (created with biorender.com)

The operators specifically segmented the anterior half of each calcification, creating semilunar segmentations. This approach was chosen to minimize the potential impact of acoustic shadowing from hard calcifications, compared to soft and fluid calcifications, where the posterior margin is clearly delineated. The segmentation process was straightforward in most cases due to the distinct borders of calcifications. A representative example can be found in the Supplementary Material. The process of followed in this study can be found in (Fig. 1).

For radiomics feature extraction, the PyRadiomics extension within the 3D Slicer software was used. An isotropic voxel size of 1 × 1 × 1 mm was selected, with a Laplacian of Gaussian filter size set to 1, and a default bin width of 25. The extracted features, categorized into original and wavelet features as well as multiple subgroups, are summarized in Table 1. This study adhered to the updated CLAIM Checklist [21] and the CLEAR checklist [22]. The completed checklists can be found in the Supplementary Material.

**Table 1:** Extracted features categorized into group (original, wavelet) and subgroup (GLCM, first-order, GLRLM, GLSZM, GLDM, NGTDM, shape)

	Type	Count	Percentage (%)
Group			
	Original	105	12.37
	Wavelet	744	87.63
Subgroup			
	GLCM	216	25.44
	First-order	162	19.08
	GLRLM	144	16.96
	GLSZM	144	16.96
	GLDM	126	14.84
	NGTDM	45	5.30
	Shape	12	1.41

### Statistical Analysis

Descriptive statistics were computed for both continuous and categorical variables in the dataset. For continuous variables, such as age and calcification maximum diameter, the mean, standard deviation, minimum, maximum, and interquartile range (IQR) were calculated. Specifically, the mean age of the patient population was determined along with its corresponding standard deviation, and the median calcification diameter was computed along with its IQR. For categorical variables, including gender, calcification location (supraspinatus, infraspinatus, subscapularis), and calcification type (hard, soft, fluid), the absolute frequencies and percentages were calculated to describe the distribution within the patient population. This provided a clear overview of the demographic and clinical characteristics of the study cohort.

To evaluate the reliability of radiomic features extracted from ultrasound images, several statistical methods were employed. Intraclass correlation coefficient (ICC) values were calculated for each feature derived from both the original images and those processed with three different histogram equalization methods: Histogram Equalization, Standard CLAHE, and Advanced CLAHE. This analysis was conducted to assess the inter-rater reliability of features extracted by two operators.

To further investigate differences across pre-processing methods, a non-parametric Friedman test was conducted, given the repeated measures design. Significant results from the Friedman test were followed by Wilcoxon signed-rank tests with Holm correction to identify pairwise differences between pre-processing methods. Boxplots were created to visualize ICC values, with significance stars added to indicate statistical differences between methods.

Group and subgroup analyses were performed to compare the consistency of various feature types, including first-order, GLCM, GLDM, GLRLM, GLSZM, NGTDM, and shape features. The Kruskal–Wallis test was applied to evaluate differences in the distribution of radiomic features across the different pre-processing methods. Additionally, the Kruskal–Wallis test was used to assess differences between calcification types and diameter ranges.

Following the Kruskal–Wallis test, Dunn’s post-hoc analysis with Bonferroni correction was conducted to identify specific pairwise differences among feature subgroups, determining which exhibited significant variations depending on the pre-processing method used. All statistical analyses were performed using the same computer configuration as previously described. A *p*-value threshold of 0.05 was set for determining statistical significance.

## Results

The study cohort consisted of 84 patients with an average age of 49.77 years (SD = 8.51), with a predominance of female participants (71.43%, *n* = 60). The majority of calcifications were located in the supraspinatus tendon, with soft calcifications being the most commonly observed type. Due to the normal distribution of the age variable, as confirmed by the Shapiro–Wilk test (*p* > 0.05), the mean and standard deviation were used for its analysis. The median maximum calcification diameter was 14.00 mm, with an interquartile range (IQR) of 9.00 mm, reflecting the variability in calcification size across the cohort.

Histogram Equalization demonstrated the fastest mean processing time (0.0003 s), followed closely by Standard CLAHE (0.0004 s), both exhibiting minimal variability. Advanced CLAHE had a higher mean processing time (0.0012 s) and variability, reflecting the complexity of its dynamic tile-grid adjustments. No significant difference in processing times was observed between Standard CLAHE and Histogram Equalization (adjusted *p*-value = 0.48), whereas Advanced CLAHE had higher processing times compared to both Standard CLAHE and Histogram Equalization (adjusted *p*-value < 0.001). Nonetheless, this slightly higher computational cost remains negligible, as the time per image is only a fraction of a second. The results are summarized in Table 2.

**Table 2:** Mean processing times (seconds) and standard deviations for each pre-processing method

Preprocessing method	Mean processing time (s)	Standard deviation (s)
Histogram equalization	0.0003	0.0002
Standard CLAHE	0.0004	0.0003
Advanced CLAHE	0.0012	0.0008

**Fig. 2:**
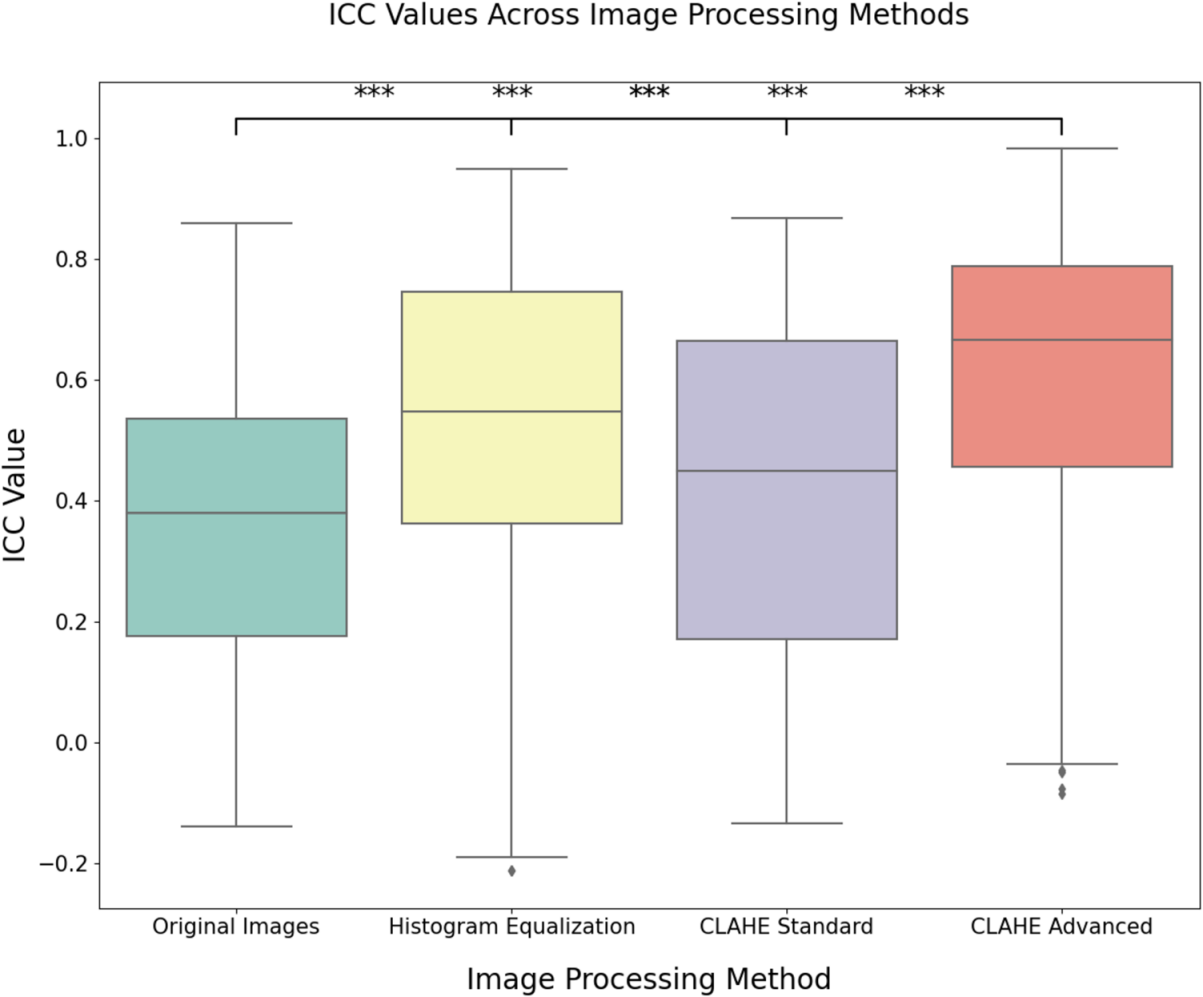
Boxplot of intraclass correlation coefficients (ICCs) for all features across the preprocessing methods. A single asterisk denotes *p* < 0.05, two asterisks represent *p* < 0.01, and three asterisks indicate *p* < 0.001

Statistical analysis revealed significant differences in the reliability of radiomic features across different image pre-processing methods (Fig. 2). The non-parametric Friedman test yielded a highly significant result (*p* < 0.001), indicating that the ICC values varied notably between the Original Images, Histogram Equalization, Standard CLAHE, and Advanced CLAHE methods. Subsequent pairwise comparisons using Wilcoxon signed-rank tests with Holm correction confirmed that all pre-processing methods significantly differed from each other.

The Kruskal–Wallis test was conducted to compare the reproducibility of original and wavelet radiomic features, using the mean ICC values across all pre-processing methods. The analysis yielded a test statistic of 0.50 and a *p*-value of 0.481, indicating no statistically significant difference in ICC values between the two groups.

**Fig. 3:**
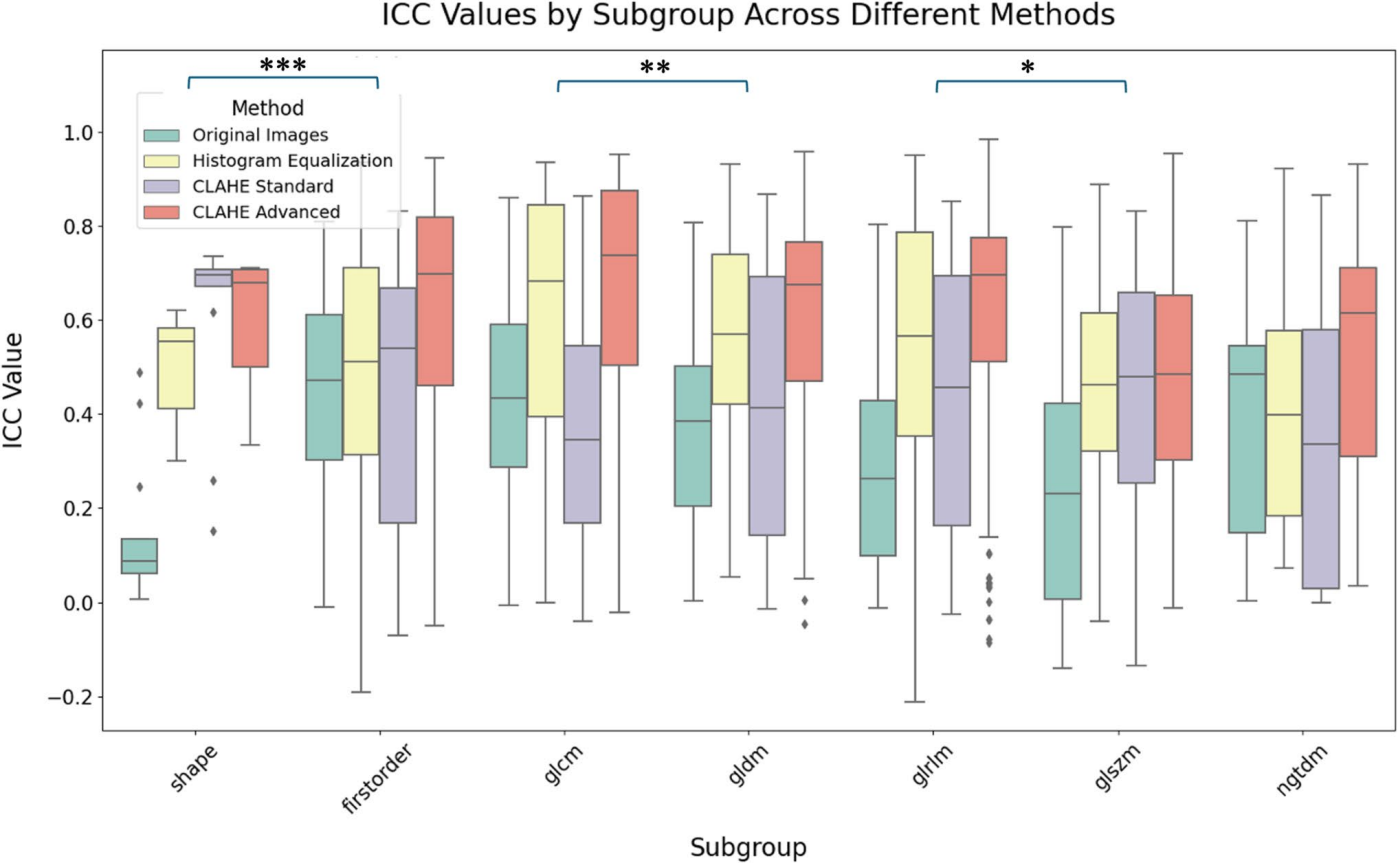
Boxplot comparison of ICCs across radiomic feature subgroups for all the preprocessing methods. A single asterisk denotes *p* < 0.05, two asterisks represent *p* < 0.01, and three asterisks indicate *p* < 0.001

Statistically significant differences in ICC values were found across most radiomic feature subgroups, with a test statistic of 96.30 and a *p*-value < 0.001 (Fig. 3). Post-hoc analysis using Dunn’s test with Holm correction confirmed significant pairwise differences, particularly involving GLCM, GLSZM, and first-order features. For example, GLCM features differed significantly from GLRLM and GLSZM (*p* < 0.001), while first-order features were significantly different from GLSZM and NGTDM (*p* < 0.001). In contrast, shape features did not show significant differences from any other subgroups. A separate analysis comparing shape features to first-order features yielded a non-significant result (*p* = 0.317), and a broader comparison to all other feature types also showed no significant differences (*p* = 0.710).

In terms of calcification types, the Kruskal–Wallis test results indicated no significant differences in radiomic feature distributions across different calcification types for any of the pre-processing methods. The *p*-values for all methods were well above 0.05, with Original Images showing a Kruskal–Wallis statistic of 0.04 and a *p*-value of 0.980, suggesting that calcification type does not significantly influence feature reproducibility.

**Fig. 4:**
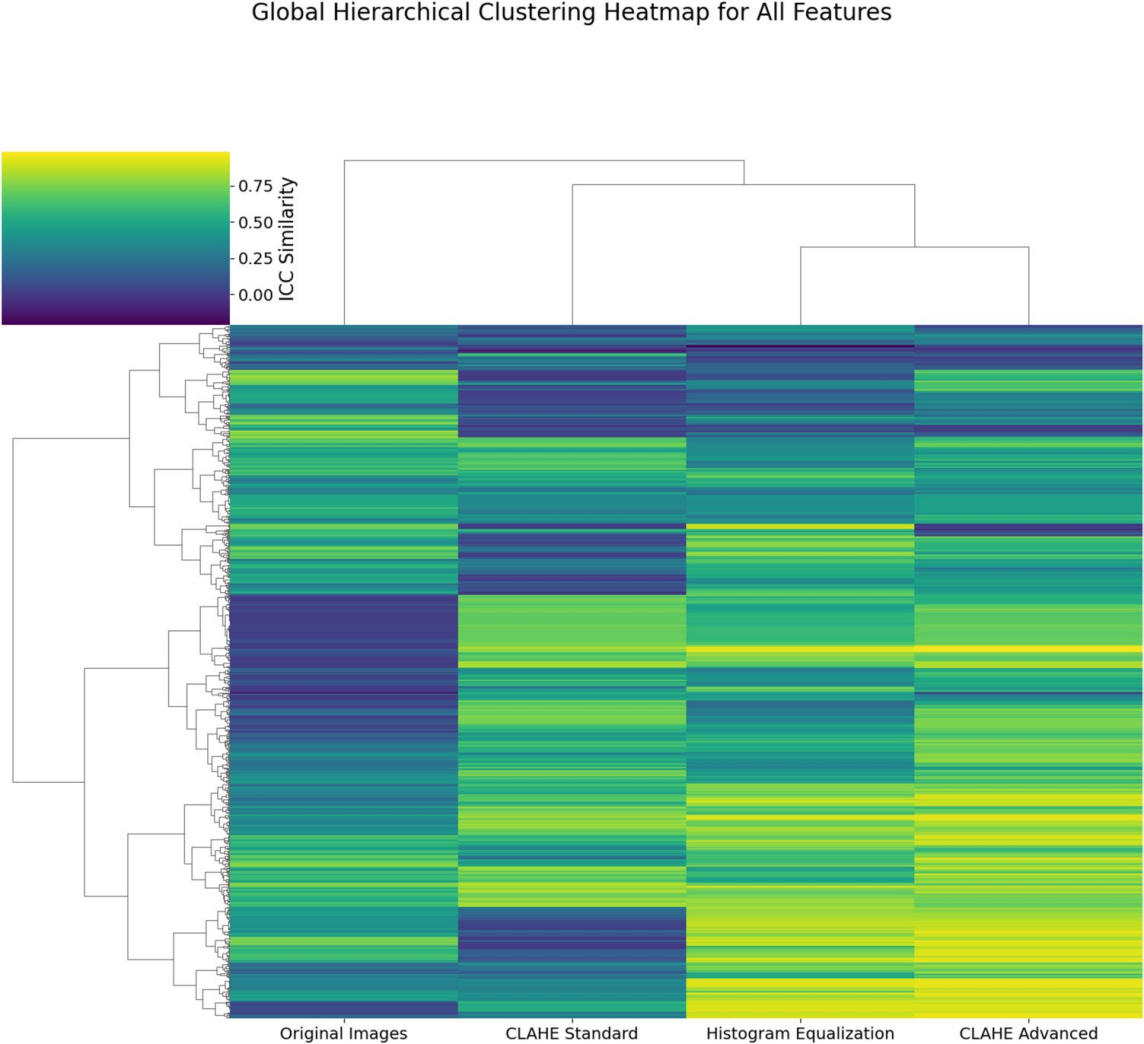
Global heatmap with hierarchical clustering of ICC values across all features and preprocessing methods

**Fig. 5:**
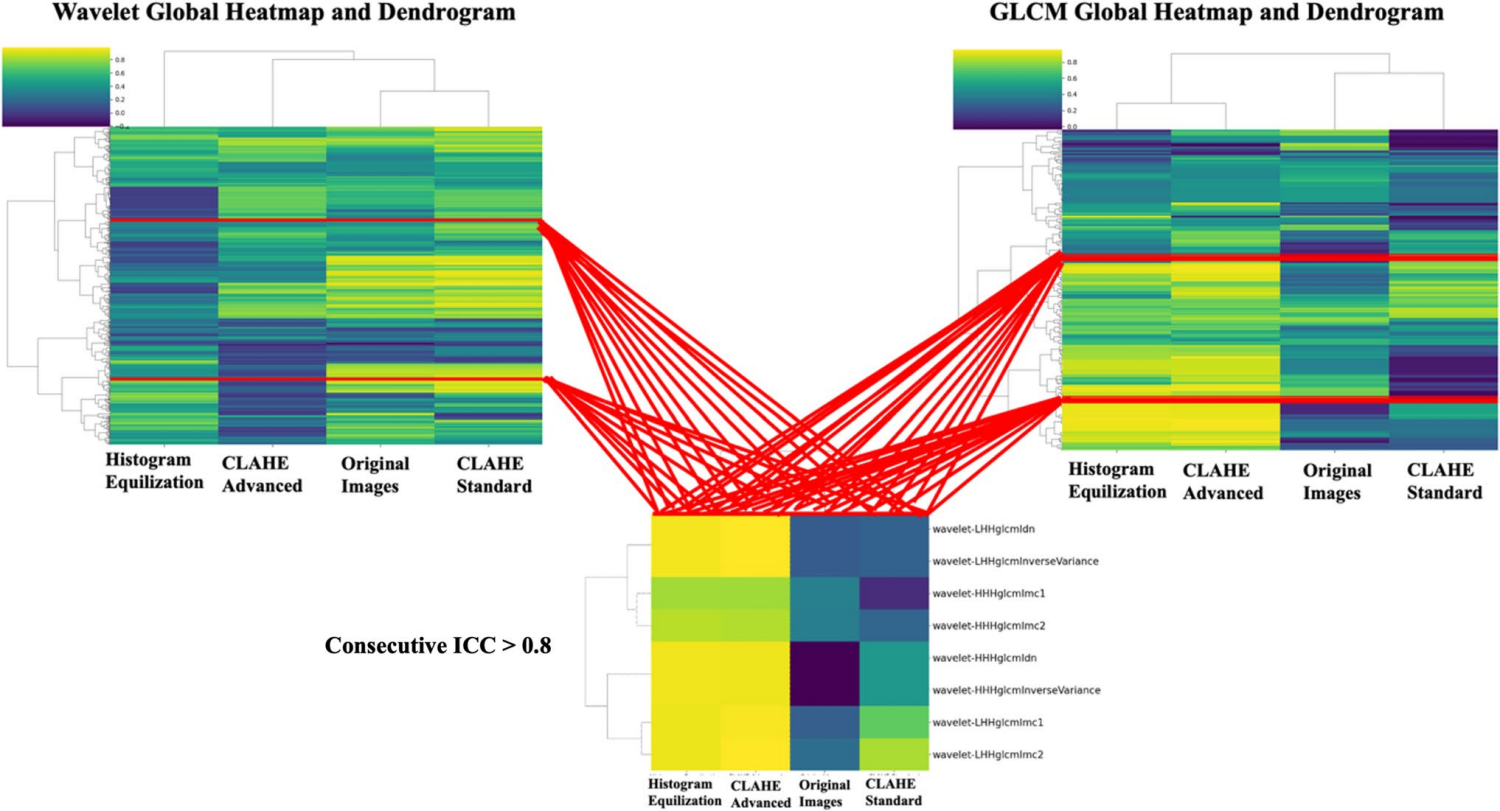
Heatmaps and dendrograms illustrating the ICCs across wavelet-based (left) and GLCM (right) features across different preprocessing groups. The bottom matrix highlights eight consecutive features identified at the intersection of wavelet and GLCM

A global hierarchical clustering heatmap with Euclidean distance was used to display ICC values across four image processing methods (Fig. 4). High ICC values indicate more reliable features, while clustering illustrates relationships in feature reproducibility across pre-processing techniques. While Original Images and Standard CLAHE show similar ICC values for many features, Histogram Equalization and Advanced CLAHE often diverge, indicating a unique impact on feature reliability. Certain features display consistently high ICC values across all methods, suggesting robustness to pre-processing, while others vary significantly, highlighting their sensitivity to specific techniques. We further investigated for consecutive features demonstrating high intra-class correlation coefficient (ICC > 0.8) across different groups and subgroups. The analysis identified eight wavelet-based Gray Level Co-occurrence Matrix (GLCM) features with ICC > 0.8 for the Histogram Equalization and CLAHE Advanced methods (Fig. 5).

**Fig. 6:**
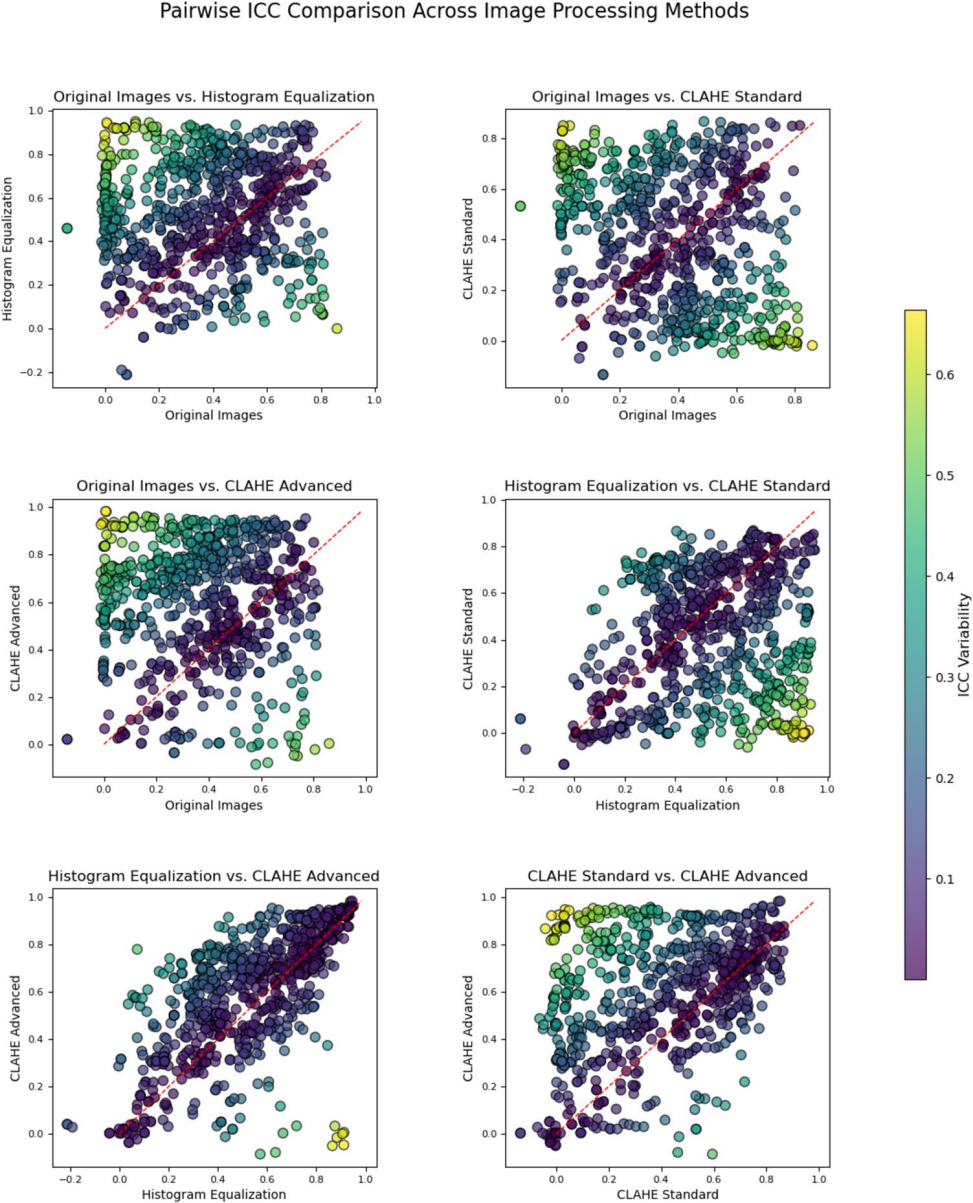
Pairwise scatterplots of ICC Values across preprocessing methods. Scatterplots illustrate ICC correlations between pairs of preprocessing methods, with points clustering along the diagonal indicating similarity in ICC values

**Fig. 7:**
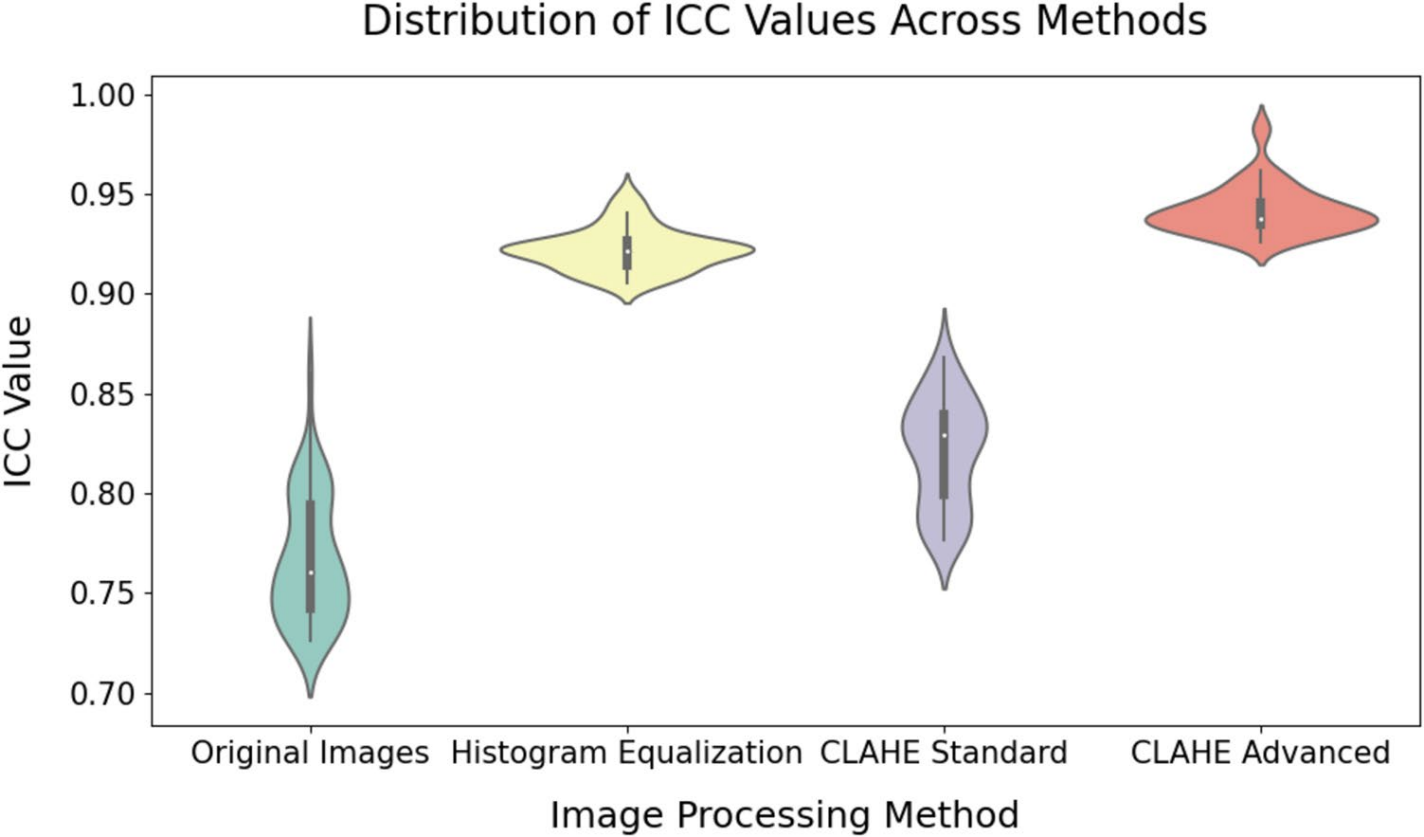
Violin plot of intraclass correlation coefficients (ICCs) for the top features across the preprocessing methods

Advanced CLAHE and Histogram Equalization produce the most similar ICC distributions, indicating comparable effects on feature reliability (Figs. 6 and 7). Following closely, the two CLAHE methods (Standard and Advanced) also show alignment, suggesting they have similar impacts on ICC values across features. This similarity within CLAHE methods highlights their consistency in enhancing contrast. Overall, the clustering near the diagonal in these comparisons suggests certain pre-processing methods yield more stable ICC values than others.

To evaluate the reproducibility of radiomic features across different pre-processing methods, the top 50 features with the highest ICC values were identified for each method: Original Images, Histogram Equalization, Standard CLAHE, and Advanced CLAHE (Supplementary Material). Among these, the Advanced CLAHE method consistently produced the highest ICCs, with features such as wavelet-LHLglrlmRunVariance and wavelet-LHLglrlmLongRunEmphasis achieving ICC values of 0.983 and 0.982, respectively. Other notable features under Advanced CLAHE include wavelet-LHHglrlmLongRunLowGrayLevelEmphasis with an ICC of 0.961 and wavelet-HHLglcmSumEntropy with an ICC of 0.953. In comparison, the highest ICC observed for Original Images was 0.860, for the feature wavelet-HLLglcmClusterProminence, while under Histogram Equalization, wavelet-LHHglrlmLongRunEmphasis reached an ICC of 0.951, and Standard CLAHE saw wavelet-LHLgldmDependenceEntropy with an ICC of 0.868.

For the top 50 features in terms of ICC, wavelet-transformed features consistently ranked among the highest ICCs across all methods, with Advanced CLAHE demonstrating the greatest consistency in feature reproducibility. Texture features, particularly those in the GLCM and GLRLM subgroups, showed substantial improvements under Advanced CLAHE, with mean ICC values reaching 0.695 for GLCM, compared to 0.429 for the same subgroup under Original Images. Overall, the statistical analysis confirmed significant differences across pre-processing methods, with Advanced CLAHE consistently leading to the most reproducible radiomic features. Dunn’s post-hoc analysis with Bonferroni correction further identified significant pairwise differences between radiomic feature subgroups, particularly between the GLCM and GLSZM subgroups, with *p*-values < 0.001. These findings underscore the sensitivity of certain features to the choice of pre-processing method.

## Discussion

Herein, we provide a comprehensive assessment of the effect of image pre-processing on ultrasound radiomics feature reproducibility. Three commonly used pre-processing algorithms are benchmarked against the reproducibility of radiomics features across various radiomics classes. Our results indicate that Advanced CLAHE is the most effective method for enhancing feature reproducibility; however, other methods such as Histogram Equalization and Standard CLAHE also yielded acceptable results with significantly higher number of reproducible features compared to unprocessed images.

The selection of a pre-processing method may depend on the specific analytical goals, with Advanced CLAHE generally providing the best balance of consistency and reliability across a wide array of features and datasets. Consistent with the findings of Radzi et al. in mammography and Ramli et al. in DWI-MRI images, CLAHE in this study effectively enhanced radiomic feature reproducibility [19, 23]. As noted in both studies, Advanced CLAHE facilitated a robust contrast enhancement that improved feature stability. CLAHE’s utility across diverse imaging modalities—including mammography [19], MRI [23], and ultrasound in this study—suggests its potential as a versatile enhancement technique applicable across various radiomic contexts. This versatility enhances CLAHE’s value in settings where reproducibility is crucial.

Though no statistical significance was observed between original and wavelet-transformed features, top feature analysis revealed that wavelet-transformed features exhibited robustness across methods. The dominance of wavelet features, particularly within GLCM and GLRLM subgroups, underscores their reliability and potential as stable biomarkers in radiomic studies. These features appear less sensitive to pre-processing variations and remain consistent across datasets, aligning with evidence that wavelet-based methods generally reduce noise impact and improve stability (Zhang et al., [24]).

Texture features showed improved stability, especially across GLCM-based, as well as GLRLM, according to Ramli and Radzi [19, 23]. First-order features came out to be more robust compared to shape features, but not compared to texture features, as Traverso suggested pooling results from multiple studies, mostly based on CT-radiomics [4].

Importantly, certain features demonstrated remarkable consistency regardless of the pre-processing method applied. These consistently high-performing features, many of which are wavelet-transformed, suggest their robustness and reliability as potential biomarkers in clinical applications. The fact that these features maintained high ICC values across different datasets and pre-processing methods further reinforce their value in radiomic analysis.

This study stands out by focusing on radiomics applied to calcifications in MSK conditions, a topic that has been underexplored despite its clinical significance. While previous research has primarily targeted calcified arterial plaques [25, 26] or breast microcalcifications [27], the approach in this study is broader, considering calcifications and calcified structures within a variety of pathological contexts, including tumours, degenerative conditions, and both benign and malignant processes. Notably, some studies even set the presence of calcifications as an exclusion criterion, underscoring the challenges and complexities associated with these structures [28]. Conversely, other studies recognize the diagnostic and prognostic value of calcification type and characteristics, incorporating them as independent clinical factors into their algorithms after demonstrating their statistical significance [29–31]. However, no other studies have applied radiomics directly to these calcified structures, making this study unique in its approach to leveraging radiomic analysis for enhanced diagnostic insights.

The use of CLAHE, particularly the Advanced version, proved effective in enhancing the reproducibility of radiomic features in this study in diffusion-weighted imaging of the cervical cancer [23]. While CLAHE has been employed in other imaging modalities, such as in the work by Ramli et al. [23] and Yu et al. [32], its application in ultrasound and benchmarking against other methods in radiomics is novel [23, 32].

This study’s relatively small dataset is a limitation that underscores the need for further research; nonetheless, CLAHE’s consistent performance even in smaller datasets suggests its reliability across contexts [19, 23]. Additionally, using a single ultrasound machine and operator could be a potential limitation, even though in the setting of our study it is extremely to limit other sources of variability such as across-vendor or operator variability in order to focus only on the effect of image pre-processing. Lastly, the absence of intra-rater repeatability analysis presents a potential limitation. Nonetheless, such analysis was deemed unnecessary due to well-delineated calcification boundaries.

While this study focused on calcifications in rotator cuff tendons to assess the reproducibility and stability of radiomic features, we acknowledge the potential of extending radiomics analysis to other tendon pathologies or even other anatomical entities. Future research could include comparisons of radiomic features across different musculoskeletal conditions, such as tendon tears, tendinosis, or inflammatory changes, to evaluate whether similar pre-processing approaches can enhance feature stability. Expanding the scope to other anatomical areas or tissue types, including non-musculoskeletal ultrasound imaging, may further validate and generalize the findings of this study.

Future studies should investigate the reproducibility of these findings across different ultrasound machines, vendors, probes, and patient populations, as well as extend the analysis to other ultrasound modalities, such as colour Doppler imaging, power Doppler imaging, contrast-enhanced ultrasound, and elastography. Expanding the dataset and incorporating more diverse clinical settings will be essential to confirm and enhance the generalizability of these methods.

## Supplementary Information

Below is the link to the electronic supplementary material.

Supplementary file1 (DOCX 1861 KB)

## Data Availability

Code used in this manucsript has been provided in GitHub. Data used in this publication can be provided by the corresponding author upon reasonable request.
