# Supplementary material for: The Effect of Ultrasound Image Pre-Processing on Radiomics Feature Quality: A Study on Shoulder Ultrasound
Source: J Imaging Inform Med. 2025 Feb 6;38(6):4184–95. doi: 10.1007/s10278-025-01421-w (PMC12701148; doi:10.1007/s10278-025-01421-w)
Supplement: Supplementary file 1 — Supplementary file1 (DOCX 1861 KB) [file 10278_2025_1421_MOESM1_ESM.docx]

**Dataset statistical analysis**

Table 1: Descriptive Statistics for Age

| Category | Mean | SD | Minimum | Maximum | 25^th^ percentile | 75^th^ percentile |
| --- | --- | --- | --- | --- | --- | --- |
| Age | 49.77 | 8.51 | 30 | 65 | 43 | 56 |

Table 2: Descriptive Statistics for Calcification diameter, Average Constant Score improvement

| Category | Median | 25^th^ percentile | 75^th^ percentile | Interquartile Range |
| --- | --- | --- | --- | --- |
| Calcification diameter | 14.00 | 10.00 | 19.00 | 9.00 |

Table 3: Frequency Distribution of Categorical Data for the Patient Population

| Category | Subcategories | Absolute Frequency | Percentage |
| --- | --- | --- | --- |
| Gender | Female | 60 | 71.43 |
|  | Male | 24 | 28.57 |
| Calcification Location | Supraspinatus | 74 | 88.10 |
|  | Infraspinatus | 7 | 8.33 |
|  | Subscapularis | 3 | 3.57 |
| Calcification Type | Fluid | 5 | 5.95 |
|  | Soft | 48 | 57.14 |
|  | Hard | 31 | 36.90 |

**Segmentation Example**

**
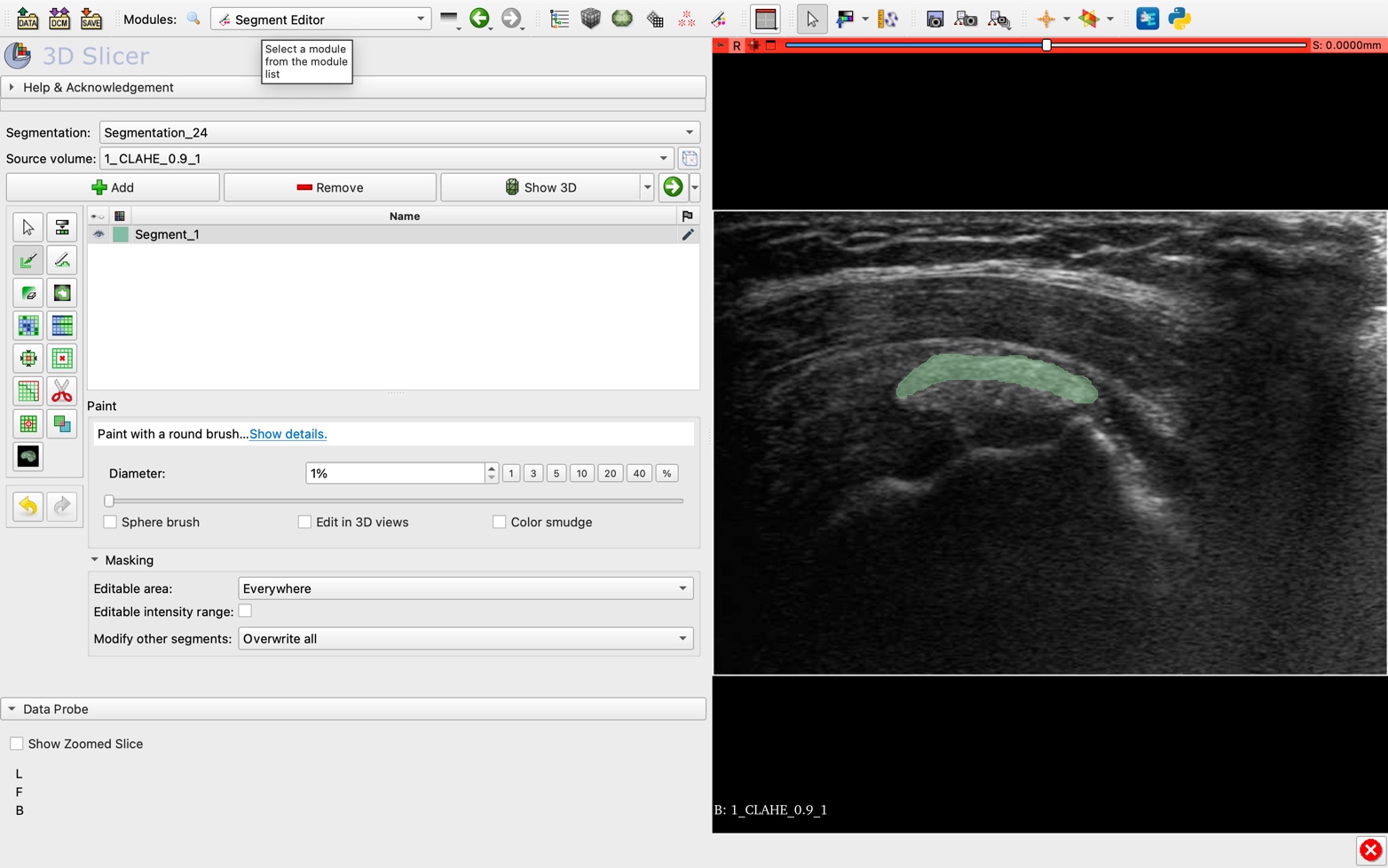
**

**3D Slicer Radiomics Extraction Framework**


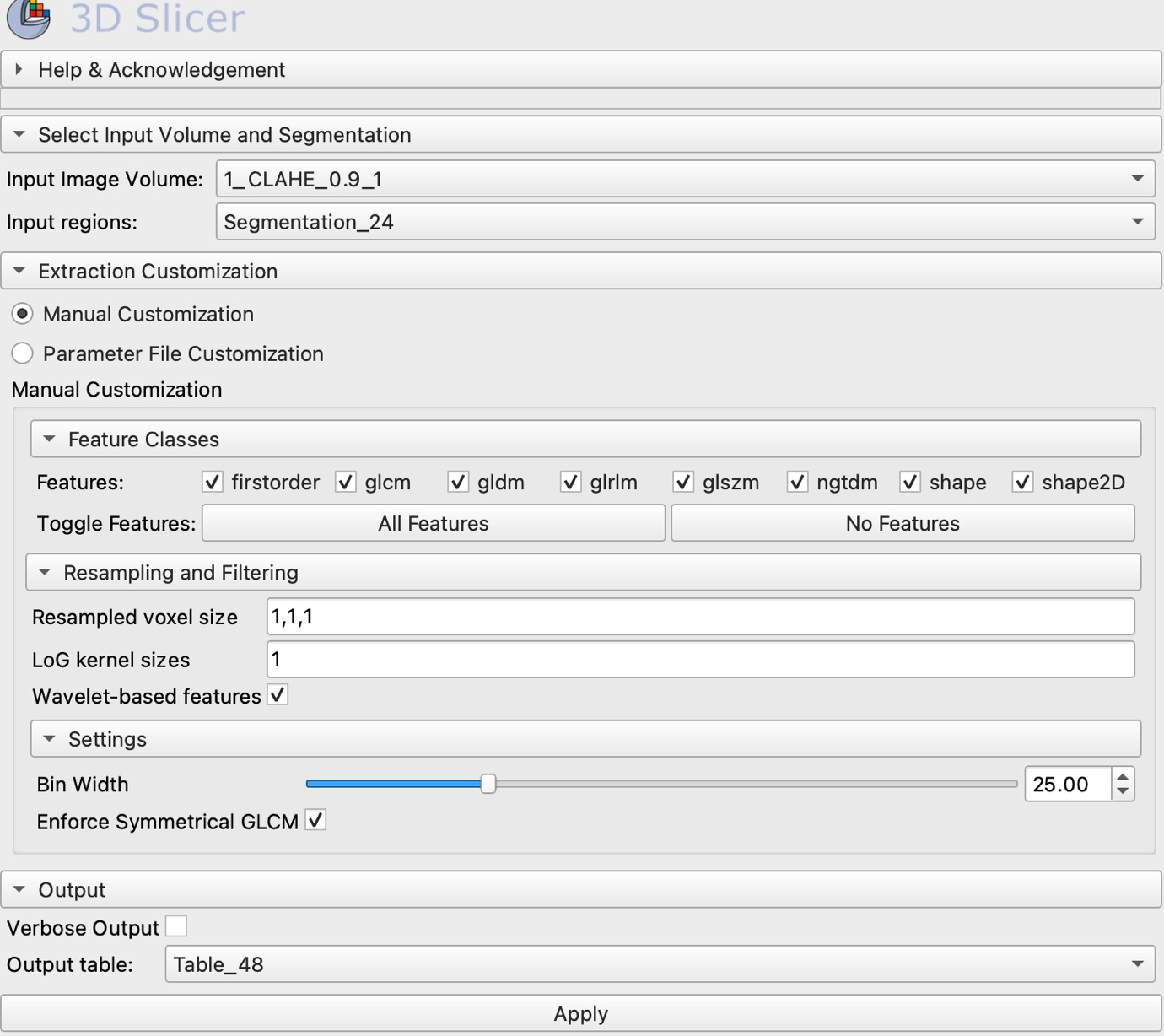


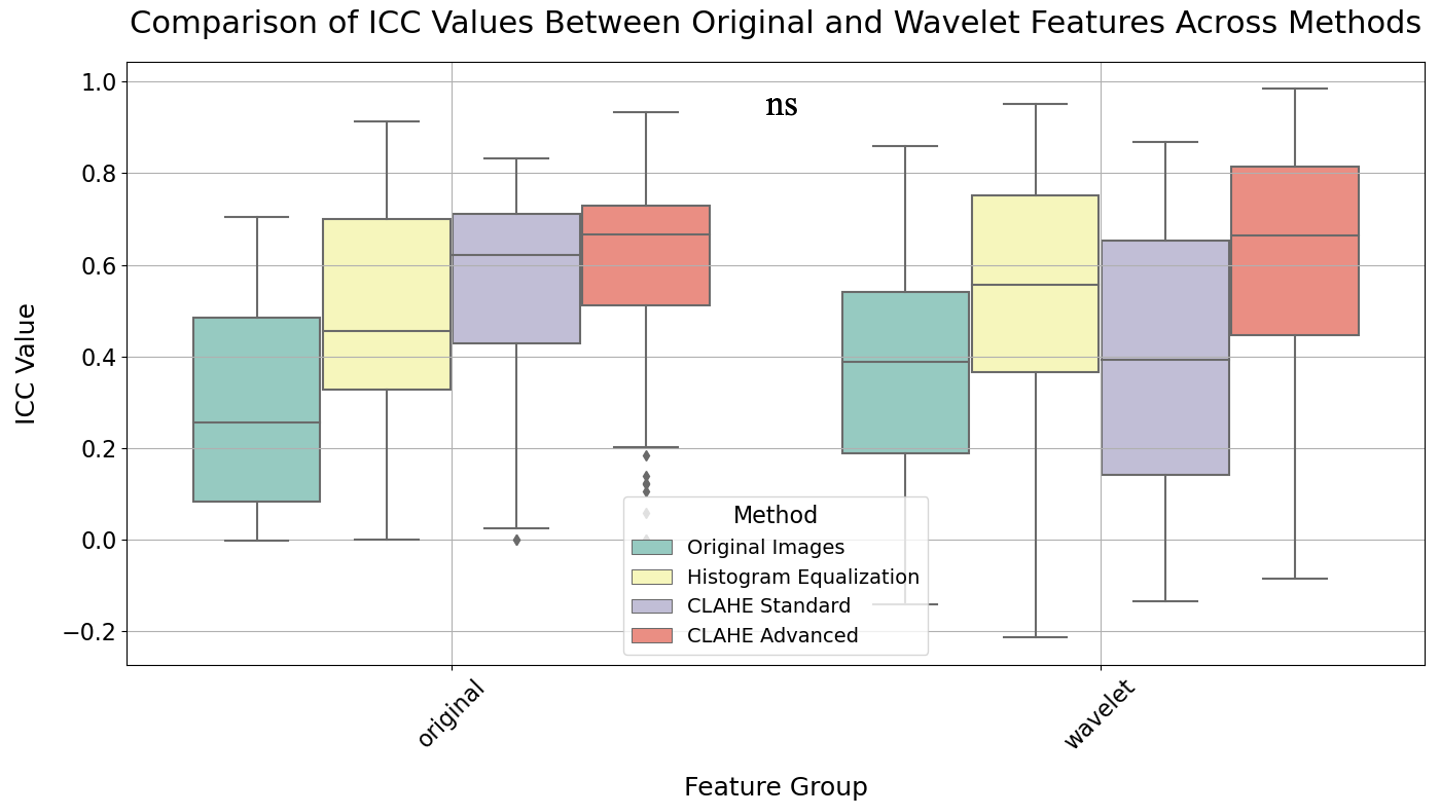


Figure 1: Boxplot Comparison of ICCs for Original vs. Wavelet-Transformed Features Across the Preprocessing Methods. "ns" denotes non-significant differences (p > 0.05).


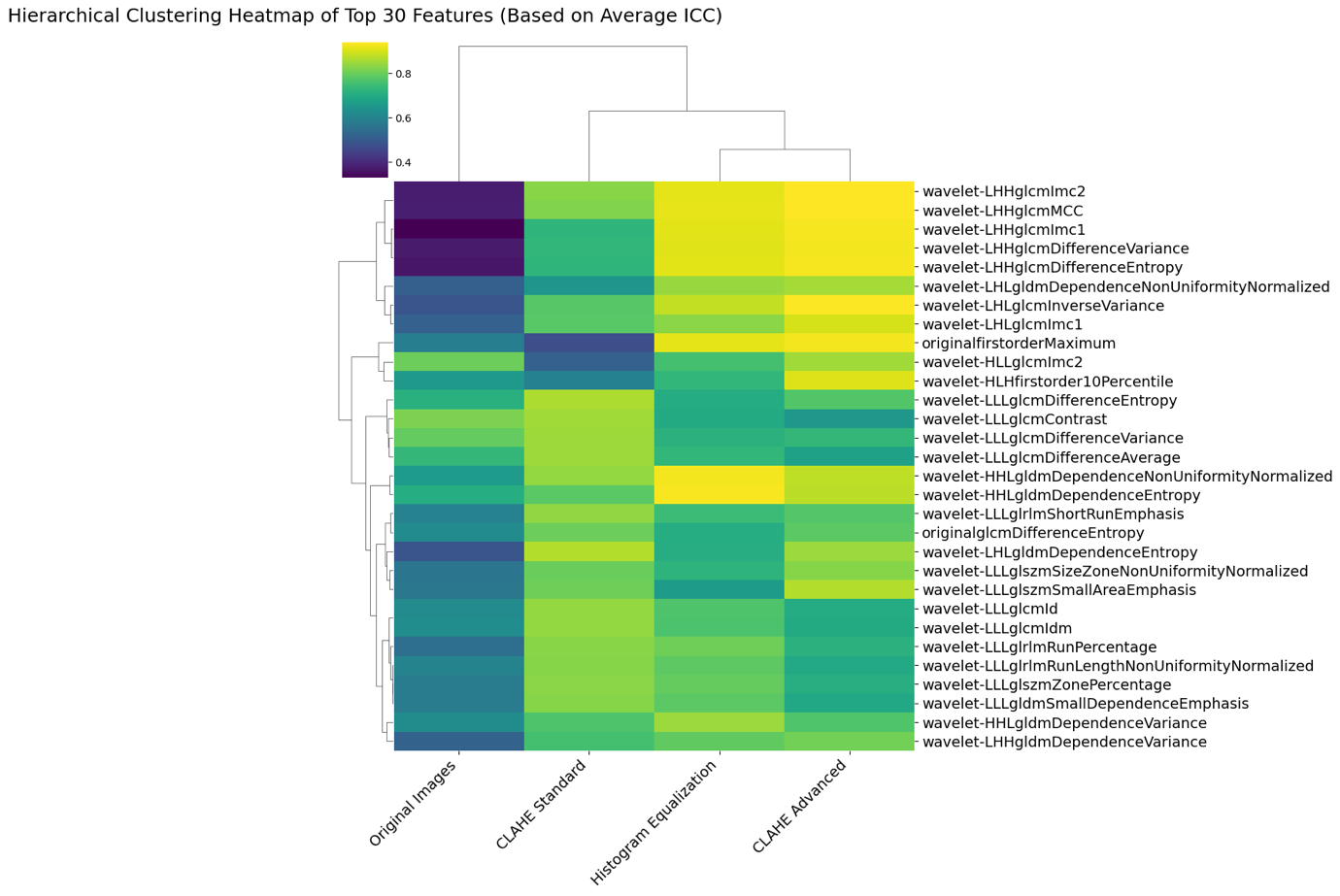


Figure 2: This hierarchical clustering heatmap visualizes the top 30 features based on ICC values across different methods.


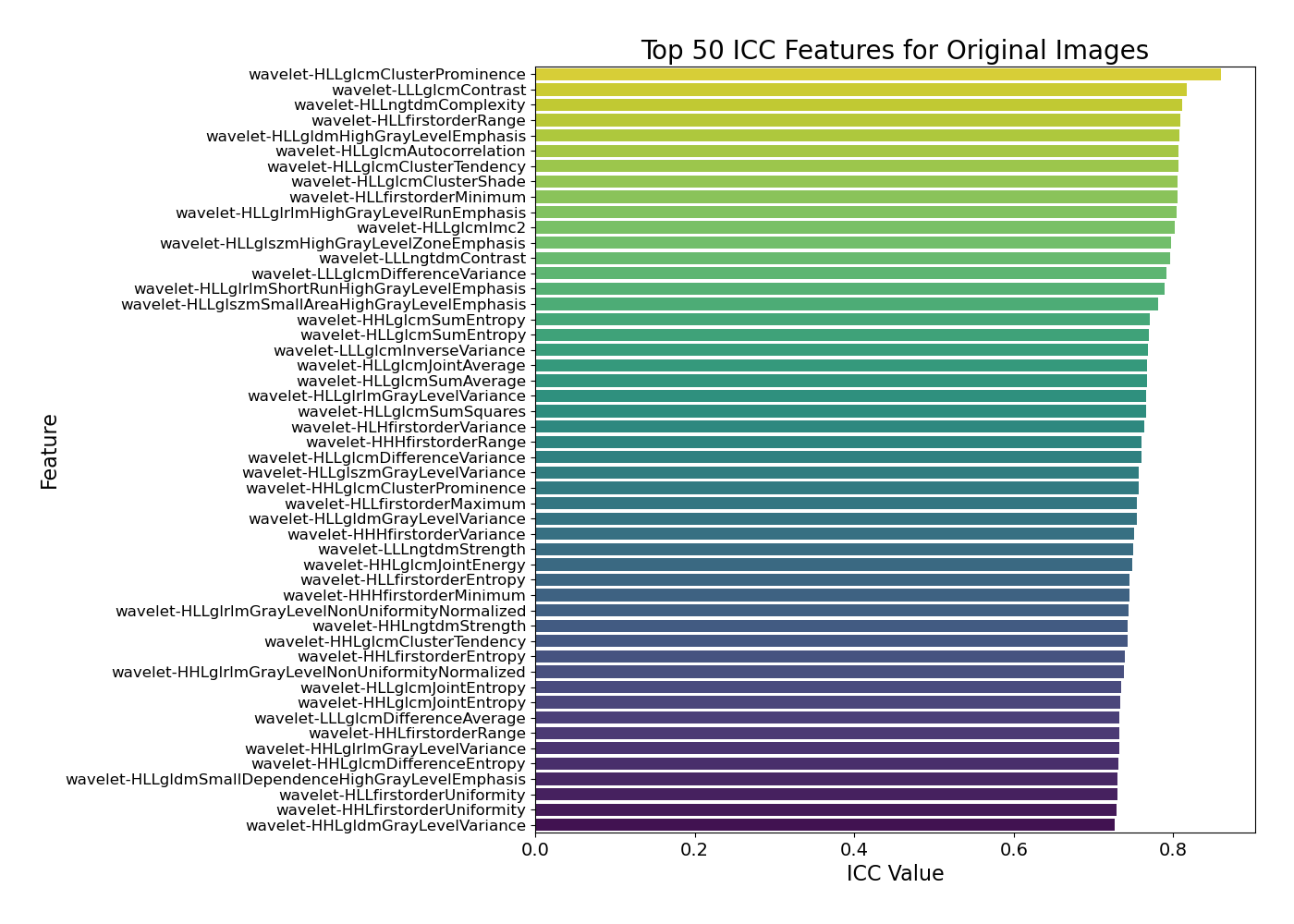


Figure 3: Top 50 Radiomic Features by Intraclass Correlation Coefficient (ICC) for Original Images.


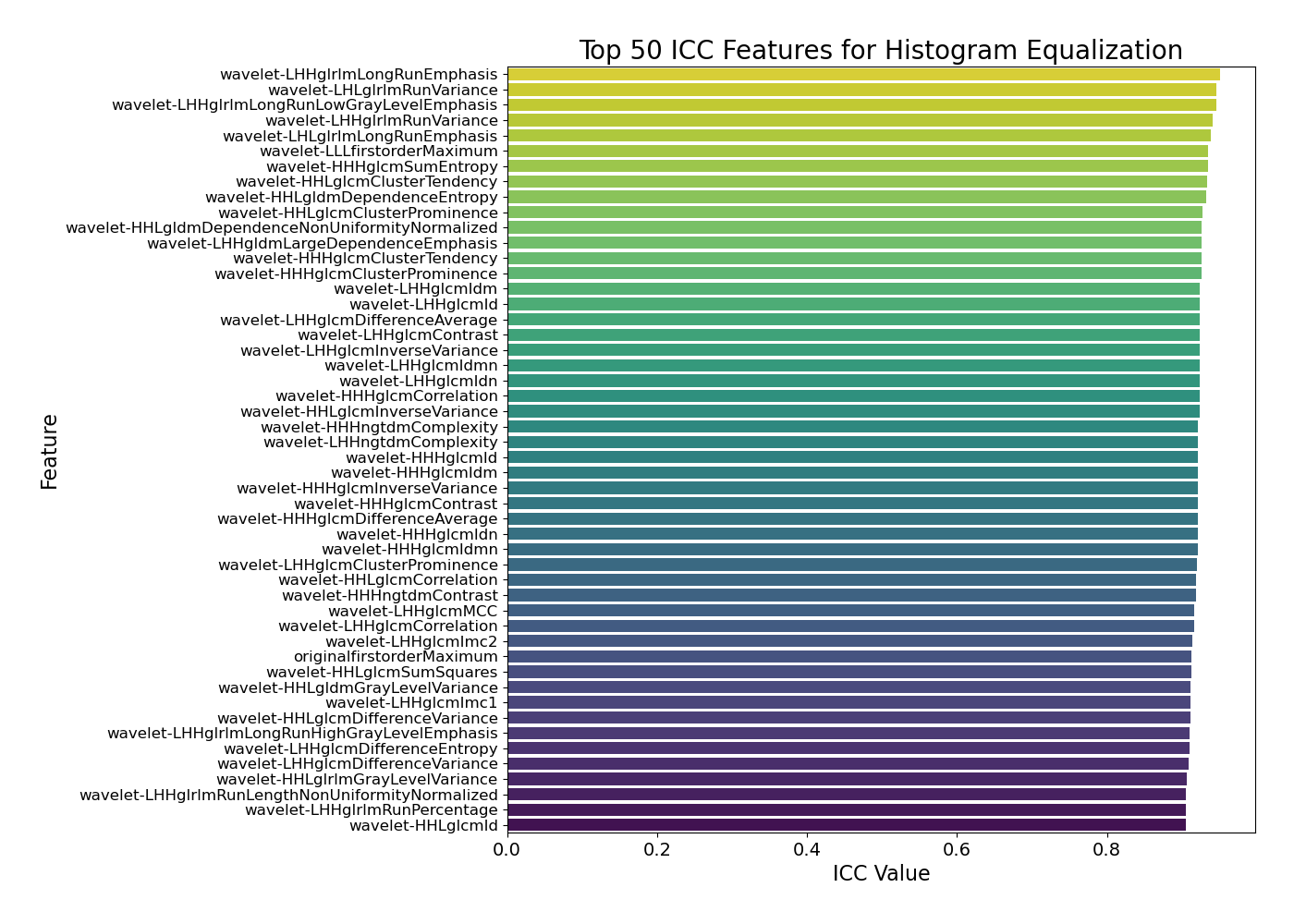


Figure 4: Top 50 Radiomic Features by Intraclass Correlation Coefficient (ICC) for Images Processed with Histogram Equalization.


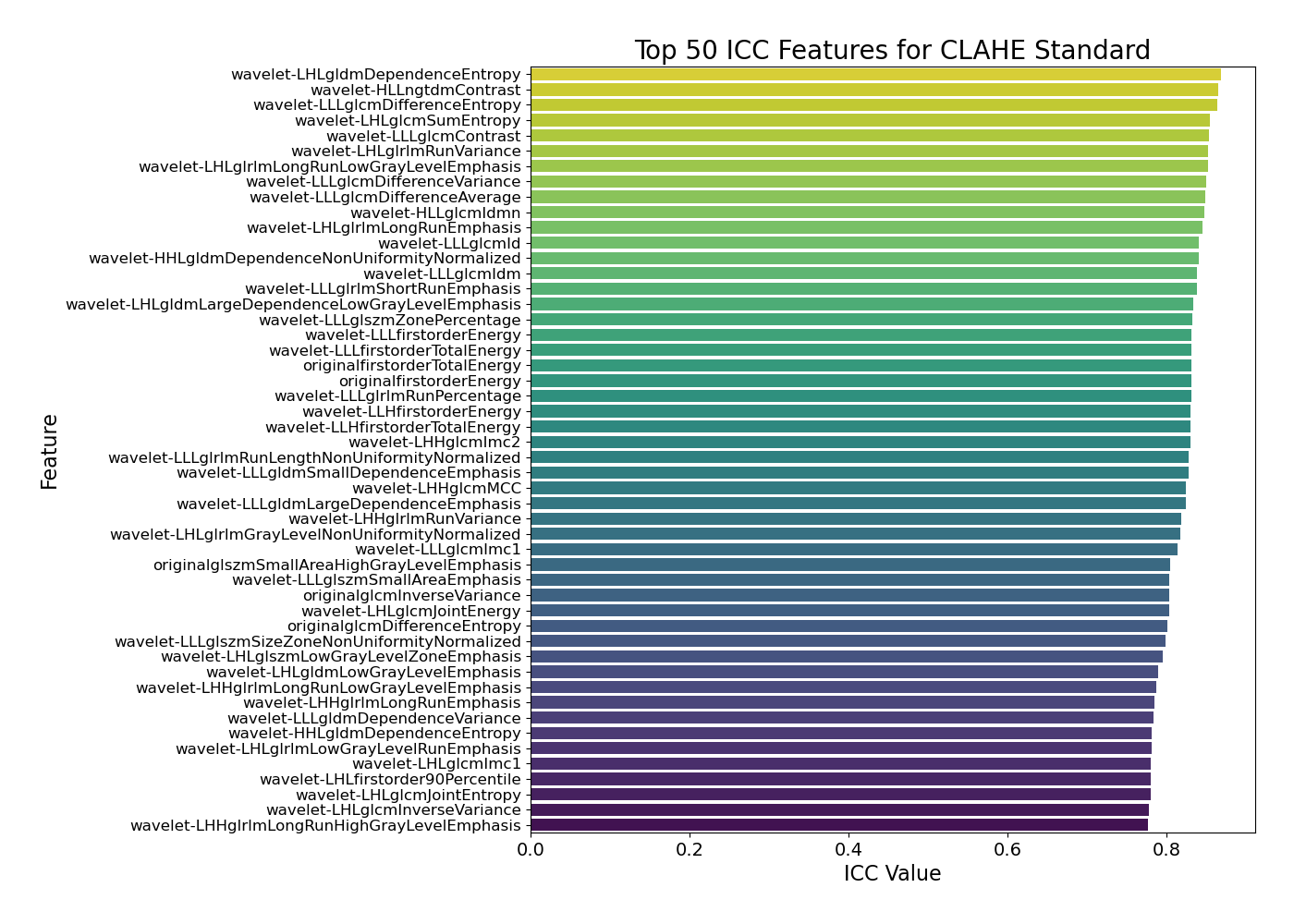


Figure 5: Top 50 Radiomic Features by Intraclass Correlation Coefficient (ICC) for Images Processed with Standard CLAHE.


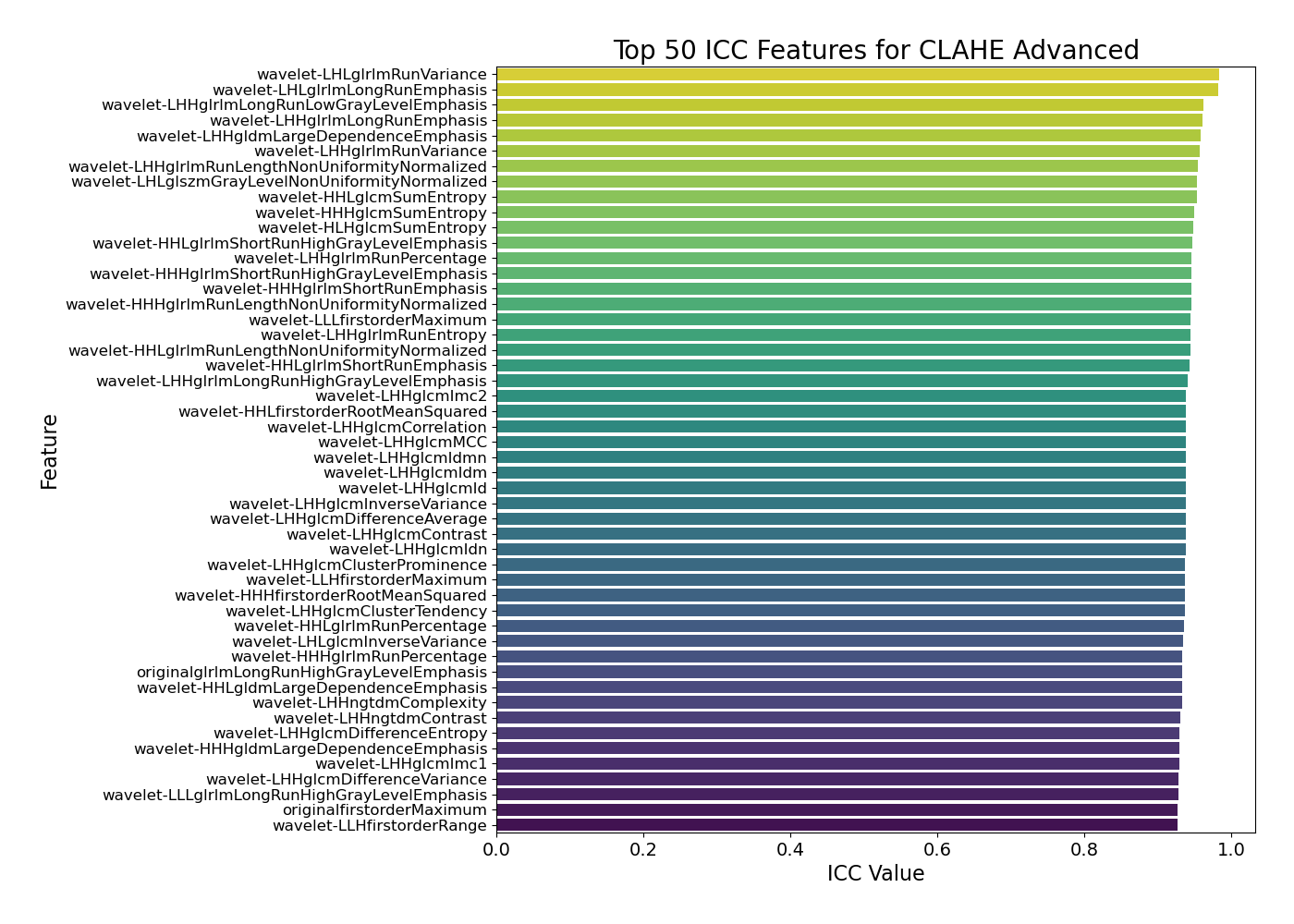


Figure 6: Top 50 Radiomic Features by Intraclass Correlation Coefficient (ICC) for Images Processed with Advanced CLAHE.

**Checklist for Artificial Intelligence in Medical Imaging (CLAIM): 2024 Update**

| Section / Topic | No. | Item | Page / Line | No | NA |
| --- | --- | --- | --- | --- | --- |
| TITLE / ABSTRACT |  |  |  |  |  |
|  | **1** | Identification as a study of AI methodology, specifying the category of technology used (e.g., deep learning) | **1/Title** |  |  |
| ABSTRACT |  |  |  |  |  |
|  | **2** | Summary of study design, methods, results, and conclusions | **2/Abstract** |  |  |
| INTRODUCTION |  |  |  |  |  |
|  | **3** | Scientific and/or clinical background, including the intended use and role of the AI approach | **3/Lines 5-11** |  |  |
|  | **4** | Study aims, objectives, and hypotheses | **4/Lines 3-10** |  |  |
| METHODS |  |  |  |  |  |
| *Study Design* | **5** | Prospective or retrospective study | **4/Line 16** |  |  |
|  | **6** | Study goal | **4/Lines 3-4** |  |  |
| *Data* | **7** | Data sources | **4/Lines 16-17** |  |  |
|  | **8** | Inclusion and exclusion criteria | **4/Lines 17-19** |  |  |
|  | **9** | Data pre-processing | **5/ Lines 2-30** |  |  |
|  | **10** | Selection of data subsets | **8/Lines 12-13** |  |  |
|  | **11** | De-identification methods | **4/Lines 26-29** |  |  |
|  | **12** | How missing data were handled | **4/Lines 17-19** |  |  |
|  | **13** | Image acquisition protocol | **4/Lines 19-22** |  |  |
| *Reference Standard* | **14** | Definition of method(s) used to obtain reference standard |  |  | **✓** |
|  | **15** | Rationale for choosing the reference standard |  |  | **✓** |
|  | **16** | Source of reference standard annotations |  |  | **✓** |
|  | **17** | Annotation of test set |  |  | **✓** |
|  | **18** | Measures of inter- and intra-rater variability of features described by the annotators | **8/ Line 1-6** |  |  |
| *Data Partitions* | **19** | How data were assigned to partitions |  |  | **✓** |
|  | **20** | Level at which partitions are disjoint |  |  | **✓** |
| *Testing Data* | **21** | Intended sample size |  |  | **✓** |

| Section / Topic | No. | Item | Page / Line | No | NA |
| --- | --- | --- | --- | --- | --- |
| *Model* | **22** | Detailed description of model |  |  | **✓** |
|  | **23** | Software libraries, frameworks, and packages |  |  | **✓** |
|  | **24** | Initialization of model parameters |  |  | **✓** |
| *Training* | **25** | Details of training approach |  |  | **✓** |
|  | **26** | Method of selecting the final model |  |  | **✓** |
|  | **27** | Ensembling techniques |  |  | **✓** |
| *Evaluation* | **28** | Metrics of model performance |  |  | **✓** |
|  | **29** | Statistical measures of significance and uncertainty | **8/Line 22-23** |  |  |
|  | **30** | Robustness or sensitivity analysis |  |  | **✓** |
|  | **31** | Methods for explainability or interpretability |  |  | **✓** |
|  | **32** | Evaluation on internal data |  |  | **✓** |
|  | **33** | Testing on external data |  |  | **✓** |
|  | **34** | Clinical trial registration |  |  | **✓** |
| RESULTS |  |  |  |  |  |
| *Data* | **35** | Numbers of patients or examinations included and excluded | **8/ Line 29** |  |  |
|  | **36** | Demographic and clinical characteristics of cases in each partition | **8/Line 29 to 9/ Line 3** |  |  |
| *Model performance* | **37** | Performance metrics and measures of statistical uncertainty |  |  | **✓** |
|  | **38** | Estimates of diagnostic performance and their precision |  |  | **✓** |
|  | **39** | Failure analysis of incorrect results |  |  | **✓** |
| DISCUSSION |  |  |  |  |  |
|  | **40** | Study limitations | **13/Line 20-27** |  |  |
|  | **41** | Implications for practice, including intended use and/or clinical role | **11/ Line 26 to 12/ Line 26** |  |  |
| OTHER INFORMATION |  |  |  |  |  |
|  | **42** | Provide a reference to the full study protocol or to additional technical details | **6/Lines 27-28** |  |  |
|  | **43** | Statement about the availability of software, trained model, and/or data | **6/Lines 7-8** |  |  |
|  | **44** | Sources of funding and other support; role of funders |  |  | **✓** |

* Indicate page and/or line number for each checklist item that is present. NA = not applicable.

**Checklist for EvaluAtion of Radiomics**

Supplementary Table S 1. CLEAR (CheckList for EvaluAtion of Radiomics research) assessment of the current study methodology. This checklist provides an overview of the radiomics reporting elements addressed in our study.

| **Domain** | **Checklist Item** | **Addressed** | **Comments** |
| --- | --- | --- | --- |
| Data | Data source(s) clearly described | Yes | Page 4, Lines 16-17 |
|  | Inclusion and exclusion criteria specified | Yes | Both inclusion and criteria specified in page 4, Lines 17-19 |
|  | Sample size justified | No | Future work could include power analysis |
| Image acquisition | Scanner(s) and acquisition protocol(s) described | Yes | Details in page 4 Lines 19-22 |
|  | Reconstruction parameters reported | N/A |  |
| Segmentation | Segmentation method described | Yes | Detailed in Materials and Methods 2.3 subsection and Supplementary Materials |
|  | Software for segmentation specified | Yes | 3D Slicer (version 5.2 for MacOS) |
|  | Intra/inter-rater reproducibility of segmentations assessed | Yes | Inter-rater analysis with ICC |
| Feature extraction | Image preprocessing steps described | Yes | Detailed in Materials and Methods 2.2 subsection |
|  | Radiomics feature extraction methodology described | Yes | Detailed in Materials and Methods 2.3 subsection and Supplementary Materials |
|  | Feature reduction or selection methods described | Yes | ICC-based stability |
| Modeling | Not applicable (no predictive modeling performed) | N/A^*^ |  |
| Evaluation | Statistical methods for feature analysis described | Yes | Detailed in Materials and Methods 2.4 subsection |
|  | Multiple testing correction applied | Yes | Holm and Bonferroni corrections |
| Biological validation | Biological interpretation of features discussed | Partially | Texture features linked to calcification type |

^*N/A: Not Applicable - indicates checklist items that are not relevant to the current study methodology.^
